# Capturing static and dynamic dietary patterns for human gut microbiome research: a conceptual framework

**DOI:** 10.1080/29933935.2026.2665578

**Published:** 2026-04-29

**Authors:** Julia S. Oliveira, Kathyrn S. Keim, Richard Evans, Jonathan Kurasch, Cyrus Jahansouz, Levi M. Teigen, Annie W. Lin

**Affiliations:** a The Hormel Institute, University of Minnesota, Austin, MN, United States; b Clinical and Translational Science Institute, University of Minnesota, Minneapolis, MN, United States; c Department of Surgery, University of Minnesota, Minneapolis, MN, United States; d Department of Food Science and Nutrition, University of Minnesota, St. Paul, MN, United States

**Keywords:** Dietary patterns, food choice, dietary assessment, gastrointestinal microbiome, personalized nutrition

## Abstract

There is inconsistency in the evidence regarding the effects of food on the gut microbiome. These inconsistencies arise, in part, from substantial inter- and intraindividual variations in diet. The wide range of foods consumed directly influences substrate availability for the microbiota. By categorizing foods into broad groups and overlooking interactions among food constituents within individual foods, current dietary pattern approaches can obscure food-specific differences needed to understand dietary effects. Differences in habitual and occasional intake further complicate analyses since frequency of food consumption can produce different gut microbiota responses within the same individual. Flexible analytical approaches are needed to capture within-individual food intake frequency and food-specific effects. To address these challenges, this narrative review presents dietary pattern concepts that distinguish static (stable or consistent) and dynamic (fluctuating or episodic) intake of specific foods at the individual level. We performed a literature search in three databases, including Medline, CINAHL, and PsycINFO, to retrieve relevant articles that distinguish the concepts of “core foods” and “secondary foods” in population-level studies. We adapt these concepts to a microbiome context at the individual level and propose future directions for studies investigating the impact of diet on the gut microbiome.

## Introduction

The gut microbiota plays an important role in maintaining health by supporting nutrient extraction and metabolism, as well as regulating the immune system.^1^^,^^2^ Disruption in the composition and function of the gut microbiota, known as dysbiosis, is associated with several adverse health conditions, including cardiovascular disease,^3^ colorectal cancer,^4^ and inflammatory bowel disease.^5^ Alterations in gut microbial composition have been linked to a wide spectrum of diseases, including metabolic, gastrointestinal, and immune-mediated conditions.^6^ Diet is a modifiable therapeutic target that may reduce microbiome-mediated disease risk through its effects on the gut microbiota.^6-8^ While several gut microbiome dietary interventions have focused on fiber intake, the overall combination of foods (i.e., dietary patterns) consumed has increasingly been recognized as contributing to gut microbiota composition and diversity.^8-10^

Recent findings suggest that gut microbiota composition is strongly associated with specific foods than with nutrient profiles.^11^ Diet-induced changes in the gut microbiome may result from changes in substrate delivered by foods and related microbial fermentation activity.^8^^,^^12^^,^^13^ The importance of examining dietary intake is evident in studies of dietary patterns and the gut microbiome. Dietary patterns such as the Mediterranean diet, Asian-style diets, high-polyphenol diets, high-fiber diets, and plant-based diets had higher abundance of short-chain fatty acid- or lactic acid-producing bacteria.^14-16^ These patterns have also been linked with reduced abundance of pathogenic bacteria.

Many specific foods appear across multiple dietary patterns, and microbiota responses may differ depending on the combination of foods consumed. This overlap of foods across dietary patterns complicates interpretation of how individual food items affect the gut microbiome and may contribute to inconsistent findings across studies, as for example in studies with red meat,^17^^,^^18^ non-calorie sweeteners,^19^^,^^20^ and non-fermentable dairy products.^21^^,^^22^ Beyond overlapping foods across dietary patterns, individuals can shift eating behaviors without adhering to a single broadly defined dietary pattern.^23-26^ Gut microbiome responses are ultimately driven by substrate made available by individual foods, making within-person variability in food intake critical for interpreting diet–microbiome interactions and identifying “responders” and “non-responders”.^27-30^

Inconsistent definitions of dietary pattern across individuals may influence food choices, with food intake often changing on a daily basis.^31^ Daily food selection also reflects multiple determinants, including cultural ideals (e.g., traditions and beliefs), personal factors (e.g., personal preferences), available resources (e.g., income, time, and cooking skills), social influences (e.g., group decision-making), and macrolevel contextual factors (e.g., economic conditions, government policies).^32^ Food palatability is an additional factor influencing food choices.^33^ The variability in dietary patterns and gut microbiome responses necessitates individual-centered, food-level analytic approaches that account for eating behavior.

Here, eating behavior refers to the individual food consumption patterns that reflect food choice and its determinants.^34^ Human eating behavior consists of both stable and fluctuating components.^35-37^ These concepts are labeled as ***static*** and ***dynamic dietary patterns*** in this manuscript, conceptualized at the level of individual food consumption rather than predefined population-level diets. Static and dynamic dietary patterns describe patterns of food intake over time. Within this framework, static patterns are characterized by a combination of foods that are consumed frequently and consistently (i.e., core foods) while dynamic patterns focus on foods whose consumption fluctuates over shorter time scales (i.e., secondary foods). We describe how “core foods” and “secondary foods” have been conventionally defined in previous studies and we introduce the concept of fringe foods. We then propose an approach to re-define static and dynamic dietary patterns at the individual level for application in diet-microbiome research. The proposed framework can help researchers and practitioners account for eating behavior and elucidate diet-gut microbiota relationships with greater granularity.

## Materials and methods

This report is a narrative review, which does not require ethics approval or registration. The *Scale for Quality Assessment of Narrative Review Articles* (SANRA) was applied in this study (SUPPLEMENTARY MATERIAL—Table S1).^38^ The primary objective was to introduce the concepts of static and dynamic dietary patterns of individual dietary intake for application in microbiome research. To address this, we asked the following research question: *“How have concepts related to static and dynamic dietary patterns been used in previous research, and how can they be applied to gut microbiome research?”.*

A search strategy was developed jointly by the first author and a librarian with experience in evidence synthesis. One author searched Medline, CINAHL, and PsycINFO databases for relevant studies using combinations of the following keywords: “static diet”, “dynamic diet”, “core food”, and “secondary food” (Supplementary Material Table S2). Reference lists of included studies were also manually screened for inclusion. Articles were screened for eligibility using the Rayyan platform and subsequently reviewed by all authors to reach consensus regarding inclusion.^39^ Studies were eligible if they met the following criteria: 1) original research analyzing core food consumption based on frequency or quantity; 2) peer-reviewed; 3) English-language full-text; and 4) published between 2000 and October 2025. Studies conducted in vitro or using animal models, as well as those relying on pre-established healthy or unhealthy food lists to define core foods, were excluded from the review. Relevant information was extracted from included studies and reviewed by all authors.

## Results

### Description of included studies

Of the 1,126 studies identified through the search, 757 were screened for eligibility after excluding for duplicates. Title and abstract screening excluded 740 nonrelevant studies that did not address the research questions. Seventeen studies were selected for full-text review. Two articles were excluded due the presence of nonrelevant outcomes (i.e., core foods were a pre-defined list)^40^ or lack of peer-review.^41^ One original paper manually retrieved^42^ was combined with its relevant protocol paper.^43^ Thus, 15 studies, described in 16 reports, contributed findings relevant to this review. ^43-57^ We also reviewed related studies not captured in the search to better understand relevant methodological approaches.^58-60^

Details for each study are described in Table 1. All studies were cross-sectional observational, while only one contained secondary data of longitudinal cohort.^44^ Three studies were conducted in United States,^45-47^ four studies were conducted in six countries of Africa,^48-51^ while other three studies involved 14 countries from Europe.^52-54^ The other studies were conducted in Mexico,^44^^,^^55^ Taiwan,^56^ and Hong Kong.^57^ Most studies included adults 18 years and older, while others did not have an age restriction.^44^^,^^49^^,^^52^^,^^56^ Four studies focused on infants and young children.^43^^,^^50^^,^^51^^,^^55^

**Table 1.** Description of study design, population and dietary assessments of included studies (*n* = 15).

Group	Reference (year)	Study Design	Population	Food Intake Method
**24-h food recalls**	Chung et al. (2025)^57^	Total Diet Study*	Adult population from Hong Kong	Two 24-h food recalls, non-consecutive
	Chiang et al. (2018)^56^	Total Diet Study*	7,580 individuals from all ages	At least one 24-h food recall
	Denney et al. (2017)^55^	Cross-sectional study	2057 Mexican children	Single 24-h food recall
	Marsh et al. (2012)^46^	Cross-sectional study	99 People with type 2 diabetes	Four 24-h food recalls, non-consecutive
**Food records**	Sirot et al. (2009)^52^	Total Diet Study*	2,058 adults and children from Europe	Seven food records, consecutive
	Taylor et al. (2005)^47^	Cross-sectional study	72 Native-American women	Four foods records, non-consecutive
**Food Frequency Questionnaire (FFQ)**	Mancini et al. (2020)^43^	Total Diet Study*	73,031 women aged between 40 to 65 years	A validated semi-quantitative FFQ (time period non-specified)
	Okoro et al. (2017)^48^	Cross-sectional study	183 construction workers in South Africa	14-item FFQ, referred to 1 week of work
**Household Budget Surveys (HBS)**	Ingenbleek et al. (2017)^49^	Total Diet Study*	72,979 households of 4 African countries (Benin, Cameroon, Mali, and Nigeria)	Food consumption based on food expenditures and food produced at home for 2 weeks, and according to the number of inhabitants.
**Qualitative approach**	Kimiywe et al. (2024)^51^	Two-phase Qualitative Study	Infants and young children from Kenya, interviews with mothers, health workers and vendors	Two days of focus group and one day for semi structured interviews, which included free listing
	Thuita et al. (2019)^50^	Qualitative Study	Care-givers of infants receiving complementary feeding from Kenya	One day of free listing and qualitative 24-h recall
**Multiple approaches**	Devlin et al. (2014)^53^	Total Diet Study*	European adults from 17 countries	One to two 24-h food recalls or three or seven food records, consecutive or non
	Devlin et al. (2014)^54^	Total Diet Study*	European adults from 14 countries	One to two 24-h food recalls or three or seven food records, consecutive or non
	Ortega et al. (2002)^44^	Secondary data analysis with 3 studies	801 individuals from all ages	Single 24-h food recalls or 4 days of 24-h food recalls + FFQ along the seasons
	Pennington et al. (2002)^45^	Cross-sectional study	16,065 American adults	Single 24-h food recall + single food record

^*^ Total Diet Study: cross-sectional large-studies designed to estimate the average dietary intake of nutrients or chemical contaminants of a specific population, based on national data

Dietary data was collected using a single assessment method or a combination of several methods, including 24-hour dietary recalls,^44-46^^,^^53-57^ food records,^45^^,^^47^^,^^52-54^ and food frequency questionnaires (FFQ).^43^^,^^44^^,^^48^ Characteristics and limitations related to static and dynamic dietary patterns of each type of dietary assessment are summarized in Table 2. Assessment frequency varied by method, with up to seven days of dietary recalls or foods records.^52-54^ Regarding FFQ, the covered time also varied between one week,^48^ each season of the year,^44^ or without a specified reference time.^43^ Other used methods were qualitative 24-h recalls with or without free listing,^50^^,^^51^ or household budget survey.^49^ Only two studies describe whether dietary data were collected on weekdays or weekends.^53^^,^^54^ Only one study evaluated difference in the consumption along the seasons of the year.^44^

**Table 2.** Summary of dietary assessments used in included studies (*n* = 15) and limitations related to static and dynamic dietary patterns.

Classification	Dietary Assessment	Characteristics	Limitations
**Short-Term**	**24-h Food Recall**	Interview-based method in which an individual reports all foods and beverages consumed in the previous 24 hours. Often conducted by a trained interviewer using probes and portion-size aids. Most popular tool used in dietary studies.	Relies on memory (recall bias); may underreport selected foods; does not capture habitual intake, unless collected for several days
	**24-h Food Record**	The participant records foods and drinks consumed over 24 hours in real time (or shortly after eating), with portion sizes, preparation, and brands when possible.	High respondent burden; may change eating behavior (reactivity bias); requires nutrition literacy
	**Qualitative 24-h Recall**	Similar to 24-h Recall but does not quantify portions; focuses only on foods and beverages consumed.	Can not estimate energy or nutrient intake; similar to limitations to 24-h recalls
		
	**Household Budget Survey (HBS)**	Uses household food purchase/expenditure data to estimate per-capita availability and approximate intake.	Measures purchases, not intake; cannot capture individual variability; pre-specified food list.
	**Free Listing**	Participants list foods commonly consumed or culturally relevant. May be focused on cultural aspects.	Does not measure actual intake; higher risk of recall and perception bias.
**Long-Term**	**Food Frequency Questionnaire (FFQ) or Dietary Screener**	Long-term retrospective questionnaire assessing how often foods are consumed over determined period of time. May be qualitative (frequency intake) or semi-quantitative (frequency + portions).	Used more for population-level dietary intake; recall bias; pre-specified food list.

### Introduction of static and dynamic dietary patterns

No studies identified in our search explicitly investigated static and dynamic dietary patterns. A recent framework has emphasized the dynamic nature of food consumption and the importance of explicitly considering time and context when investigating diet-health associations.^31^ Our review builds on this foundation by offering a food-level conceptual framework of eating behavior that can be directly applied to empirical data. Understanding dietary dynamics also requires consideration of dietary stability, conceptualized here as static dietary patterns. Within this context, the concept of core foods provides a useful representation of dietary stability. Although none of the included studies examined the concept of core foods at the individual level, they collectively contribute to the definition of static dietary patterns.^43-57^ Consistent patterns emerge in the types of foods consumed daily or frequently within specific groups or populations across these studies. These consistently consumed foods can be considered an individual’s static dietary pattern and reflect consolidated decisions regarding food type, timing, and frequency of consumption. This conceptualization of static dietary patterns is consistent with principles from Sobal and Bisogni’s food choice process model.^32^ The model emphasizes that food choices evolve through repeated decision-making influenced by personal, social, cultural, and resource contexts. These decisions consolidate into routinized eating practices that support daily life and reduce food-related decision burden over time.

In our framework, static dietary patterns reflect stable food choices over time, characterized by core foods that are eaten frequently and consistently. Conversely, dynamic dietary patterns consist of secondary foods, which are consumed less frequently and/or consistently depending on the context. This pattern may exhibit cyclical consumption (e.g., foods often consumed only on weekends) or occasional consumption. This variability reflects the ongoing food choice processes described by Sobal and Bisogni;^32^ eating decisions are continually negotiated in response to changing routines, social situations, and constraints. Common triggers in this dynamic consumption can include acute events such as changes in stress levels, seasonal variation (e.g., availability of harvest, temperature of dishes), and economic factors (e.g., discounts at grocery stores). Notably, repeated exposure to similar contexts can shift secondary foods into more regular or static consumption, reflecting the dynamic and time-dependent nature of eating behavior described by Taylor et al.^31^ Capturing this variation requires dietary assessment methods that measure time and context surrounding eating behaviors.^61^

We also introduce the concept of ***fringe foods***, defined as foods that are rarely consumed or present in one’s food environment but not consumed by the individual. Fringe foods may reflect limited availability, cultural or contextual constraints, such as holiday foods, or deliberate avoidance. Accounting for fringe foods can help differentiate true non-exposure from low-frequency or constrained exposure. These patterns often stem from food intolerances, allergies, or taste preferences. This concept expands upon the idea of episodically consumed foods, which is typically used in population-level research to correct for skewed nutrient intake estimation due to multiple zeros.^62-64^ However, fringe foods also have clinical significance in gut health as they can represent a relevant class of gastrointestinal symptom triggers or behavioral barriers that the clinician should understand prior to providing a dietary intervention. This distinction provides deeper understanding of one’s food environment and potential intervention targets to improve gut health.

Previous studies have proposed similar concepts, such as habitual versus episodic intake, weekday-weekend variation, and dietary stability indices.^26^^,^^36^^,^^63^^,^^65-67^ Each concept captures a different aspect of diet stability and variability but differs from the static/dynamic dietary pattern framework. Habitual intake typically refers to the long-term average diet over weeks or months, while episodic intake describes foods that are not consumed daily. Statistical methods such as the National Cancer Institute method, SPADE, and gamma-based models have been widely used to estimate habitual intake by reducing within-person variability and characterizing population-level intake distributions.^62^^,^^63^^,^^68^ In contrast, the static and dynamic dietary pattern framework focuses on within-person rather than population-level estimation. Core foods form the stable foundation of an individual’s diet, while secondary and fringe foods capture foods that vary across days. This distinction is especially relevant for questions such as gut microbiome exposures, which depend on repeated consumption of specific foods and differ between individuals. Further, the binary distinction between habitual and episodic intake does not capture physiological or psychological aversions that may systematically exclude certain foods from an individual’s diet.

A similar consideration applies to weekday-weekend variation, which relies on a binary comparison of foods consumed on weekdays versus weekends. Static and dynamic dietary patterns may identify weekday-weekend differences, but our framework also uses a sliding window across days to avoid this dichotomy. Dietary stability indices, like our proposed framework, assess the stability of dietary patterns over time and evaluate if day-to-day variation occurs at the food-group level. However, these approaches rarely identify the specific foods responsible for that variability or distinguish between foods eaten daily and those consumed intermittently. The static and dynamic dietary pattern framework therefore offers advantages for gut microbiome research.

### Application of static and dynamic dietary patterns in prior research

**Figure 1.**
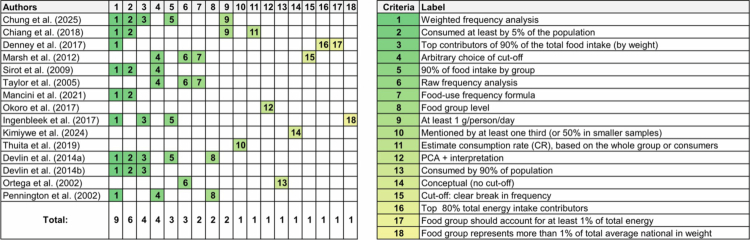
Summary of the criteria and cut-offs applied by the included studies to define “core foods,” including frequency thresholds, contribution to total intake, and minimum consumption levels.

Applying concepts of static and dynamic dietary patterns in empirical research requires consideration of how core and secondary foods are defined and measured. The included studies illustrate how prior research has defined core and secondary food concepts, all of which characterize intake at the population level (Figure 1). Two studies applied the Reaburn et al.^69^ food-use score, ranging from 20 to 100, with higher scores representing greater consumption frequency.^46^^,^^47^ The authors quantified core foods based on how often foods were consumed during a 4-day observation period. For each food, the proportion of participants was determined for each number of days the food appeared in the diet (none, one, two, three, or all observation days), resulting in five consumption categories. Each category was assigned an increasing weight, with the lowest weight assigned to foods not consumed on any day (weight = 1) and the highest weight assigned to foods consumed on all four days (weight = 5). This weighting scheme reflected day-level frequency of intake and did not account for portion size. Participant proportions were multiplied by their corresponding weights, summed across categories, and divided by five to generate a mean composite score for each food. Core foods were then identified using ranked frequency thresholds of the composite score, such as the top 20 foods^46^ or top 30 foods.^47^ Secondary foods were determined to be the subsequent 23 foods for both studies. This algorithm-based approach produced a restricted list of foods that was subsequently used in analyzes to examine the contribution of core and secondary foods on dietary intake for different populations.

Most of the included studies were conducted within the context of Total Diet Studies, a risk assessment approach used to evaluate population-level exposure to hazardous substances.^43^^,^^49^^,^^52-54^^,^^56^^,^^57^ Total Diet Studies are commonly designed to screen for chemical contaminants and estimate dietary exposure to potentially harmful compounds.^70^ A representative list of core foods is needed in this study design to characterize the total diet at the population level, given the difficulty of analyzing all foods consumed in diverse diets.

After the food list was formed in decreasing intake, the authors applied different criteria to determine the core foods across studies, with some studies using a combination of approaches, including 1) foods consumed at least by 5% of the population; 2) foods contributing to 90% of the total food intake by weight of total intake; or 3) foods providing at least 1g/person/day of each food. Some studies also applied algorithms of mean food intake to account for sampling characteristics such as age,^53^^,^^56^, sex,^56^ region,^49^ and/or consumption rate based on whole group or consumers only.^56^

There was no mention of secondary foods in any of Total Diet Studies. Only one study had thresholds, with a cutoff of regional top 30 foods and national as top 90 foods.^52^ Differences in definitions of core foods between Total Diet Studies and previously described food studies stem from their differing objectives. Total Diet Studies prioritize comprehensive dietary representation for exposure assessment and use less stringent core food definitions, while other core food studies focus on clinical contexts and diet–health relationships.

The remaining studies included in the review adopted data-driven^48^ or qualitative approaches.^50^^,^^51^ These methods were frequently applied in studies conducted in Africa and included techniques such as principal component analysis and free listing. Criteria used to identify core foods varied and included foods mentioned by at least one-third of the sample (or 50% in smaller samples), while some studies did not specify cut-off thresholds for core foods. Secondary foods were considered as culturally important foods but less salient than “core food” ^51^ or more variable in use and form, despite being widely consumed.^48^ These qualitative approaches are well suited to capturing culturally relevant foods omitted from nutrient analysis software, particularly in diverse or under-documented food environments. Interviews and focus group discussions revealed beliefs and faiths in specific foods suitable for specific groups, such as infants, which may differ from the other people in the household. The lack of standardized criteria and reliance on population-level thresholds may limit comparability across studies and constrain application to individual-level dietary assessment.

### Capturing individual-level static and dynamic dietary patterns for microbiome research

The approaches described in this review highlight the strengths of population-level classifications while underscoring the need for an individual-level framework tailored to microbiome research. Classification of a food as core or secondary within an individual’s diet cannot be inferred from summed counts of consumption frequency alone. Two individuals may consume the same food daily over a fixed period, resulting in an identical number of consumption episodes. Yet differences in daily eating occasions food pairings result in varied digestion and absorption kinetics and microbial substrate exposure. Consequently, the same food, even if in the same quantity, can have distinct metabolic effects and microbiome responses across individuals.^27-30^ Johnson et al.^11^ provides a rigorously designed example of the value of dense food-level dietary data, demonstrating that the next-day gut microbiome composition can be predicted from current-day food intake and microbiome profiles. Reducing dietary data to nutrients can mask the detection of true intervention effects or associations between food choices and the gut microbiome, and associated diseases.^6^

Achieving this level of temporal and biological resolution requires intensive data collection, which can limit feasibility in large or long-term studies. By distinguishing between static and dynamic dietary patterns, researchers can capture relevant individual nuances while reducing participant burden associated with repeated measurements. Five research considerations should be addressed through this application: 1) methods for dietary data collection; 2) duration and frequency of dietary assessment; 3) how to collapse similar foods into food groups; 4) approaches for categorizing foods into static and dynamic dietary patterns; and 5) the methods used to evaluate which substrates survive digestion and absorption to reach the microbiota.

The first consideration in nutrition-related gut microbiome research is the selection of dietary assessment instruments capable of collecting dietary exposures associated with changes in the gut microbiome. Dietary assessment instruments are commonly classified as long-term or short-term methods based on the reference period (Table 2). Food frequency questionnaires and dietary screeners assess usual dietary intake over longer periods such as the previous week, month, or year.^71^ These long-term methods are not intended to capture short-term dietary variation or timing of food intake. Rather, validated long-term dietary assessment methods describe overall dietary patterns or longer-term dietary exposure of the study sample or population.^72^

Short-term dietary assessment methods, such as 24-hour dietary recalls and food records, collect intake data for a single day and can be administered repeatedly across multiple days.^73^ The repeated-measures design captures between-day variability in individual dietary intake, a critical component for distinguishing static and dynamic dietary patterns. These repeated observations allow characterization of dietary variability within individuals and, when appropriate, may also support aggregation of shared dietary pattern classes in a study sample. Both 24-hour dietary recalls and long-term dietary assessment methods rely on participant memory and are subject to recall bias.^71^^,^^74^ Food records reduce recall bias by asking participants to document intake at the time of consumption, but this approach increases participant burden and can compromise data completeness and quality.^71^ The most common approaches are analyzing two to three dietary recalls, and up to seven foods records at the population-level. For individual-level analyzes, more repeated recalls and records may be required to capture within-person variability balanced with feasibility.^75^ For aggregate analyzes, methods such as clustering or mixed-effects models can account for inter-individual variability when comparing dietary patterns across individuals.

Secondly, frequency of data collection must be balanced against participant burden; further research is needed to determine the number of assessment days needed to accurately capture both static and dynamic diet patterns. In addition, there is currently no clear consensus regarding the amount and duration of dietary data collection required to detect dietary effects on the gut microbiome using conventional assessment tools. New dietary assessment approaches are actively being developed and evaluated outside of gut microbiome research. Technology-assisted dietary assessment methods, including electronic food records and image-based food logging, have been shown to reduce participant burden compared to pen-and-paper methods.^76^ These approaches depend on the rigor of underlying food composition databases, which may introduce additional sources of error. Emerging applications of artificial intelligence, such as image recognition for food identification, can further reduce participant burden but require additional validation and methodological development. Researchers must balance data quality, resource constraints, participant burden, and study objectives when selecting dietary assessment methods appropriate for the dietary exposure of interest in diet-microbiome research.

The third consideration involves decisions about food categorization, specifically determining which food items can be grouped together without masking biologically meaningful differences (e.g., collapsing green and black tea into a single “tea” category). Collapsing foods into broad groupings can obscure variations in microbial exposure and function. The degree of food aggregation varies substantially across nutrition studies, with dietary data condensed into as few as nine or as many as 99 food groupings depending on the level of dietary detail available. Differences in food grouping strategies complicate comparisons between studies focused on specific dietary compounds and limit the ability to identify food-specific effects on the gut microbiome. Currently, there is no consensus in gut microbiome research regarding optimal food grouping strategies. We propose to define food categories based on the specific dietary exposures of interest and to structure food groupings to preserve biological relevance. These food groups may differ by research questions or target microbiome-mediated disease. For gut microbiome research, food groupings can reflect substrates that reach the colon when the goal is to examine microbial composition or metabolic activity. Examples include fiber-rich foods (e.g., whole grains, vegetables, and fruits), fiber type or resistant starch sources, polyphenol-rich foods (e.g., berries, tea, and coffee), fermented foods (e.g., yogurt and kefir), and protein-rich foods.

Once food groupings are defined, the fourth step is to identify how these foods are prepared and consumed within individuals over time. Classifying foods as core or secondary will identify foods that contribute consistently to dietary exposure from foods consumed more intermittently or contextually. This distinction is critical for interpreting diet-microbiome interactions, as foods consumed frequently and consistently may impact baseline microbial structure, while less regularly consumed foods may contribute to short-term variation or context-specific responses.

The fifth consideration addresses the missing link between dietary intake and colonic availability: the methods used to evaluate which substrates survive digestion and absorption to reach the microbiota. Digestion kinetics is not a fully predictable process. Substrates available to the microbiome depend on several factors, including the food matrix, food physicochemical properties, and processing, as well as individual characteristics (e.g. transit intestinal time and stress).^77-80^ Further, lag effects in diet–microbiome interactions complicate interpretation of dietary effects. This variability creates several challenges to determine the exact food substrates that reaches the microbiome, thus complicating interpretation of how the gut responds to a given food based on oral intake alone. One approach to address lag effects is to evaluate the association between dietary exposures and gut-related outcomes using sliding time windows of defined length. Such approaches can reveal how specific foods or dietary patterns affect downstream microbial or gastrointestinal responses over time. Currently, no standardized recommendations exist regarding the duration (2-3 days, overlapping) or the observation window (e.g. 2 weeks) required to detect the impact of food on GI outcomes. Future pilot studies should consider evaluating varying assessment periods, while accounting for participant burden to improve tracking feasibility.

Recent approaches have been developed to infer diet from stool samples, such as FoodSeq and Metagenomic Estimation of Dietary Intake (MEDI), using either 16S rRNA gene analysis or shotgun metagenomics.^81^^,^^82^ These methods use sophisticated techniques, such as DNA metabarcodings and detection of food-derived DNA in human fecal metagenomes. Pre-defined genes from consumed foods are sequenced in stool samples using plant and animal markers genes in FoodSeq. MEDI, in contrast, maps metagenomic reads from stool against a reference library of food-derived DNA. Rather than capturing all consumed foods, these techniques identify the specific food-derived DNA that has successfully transited to the colon, providing a molecular proxy for foods that reach the microbiome. Notably, however, this approach is highly susceptible to survivorship bias as reliance on stool sampling (i.e. the substrate not used by the gut microbes) may lead to flawed conclusions about what substrate was used and made available to the microbes.

In a clinical setting, food choice is the primary target for intervention. While colonic substrates provide a biological target, altering the type or quantity of food consumed remains the first-line approach to improving gastrointestinal symptoms. The Static and Dynamic dietary pattern framework provides an upstream analysis of food intake that influences the functional capacity of the gut microbiome and identifies potential targets of change for researchers and clinicians.

**Figure 2.**
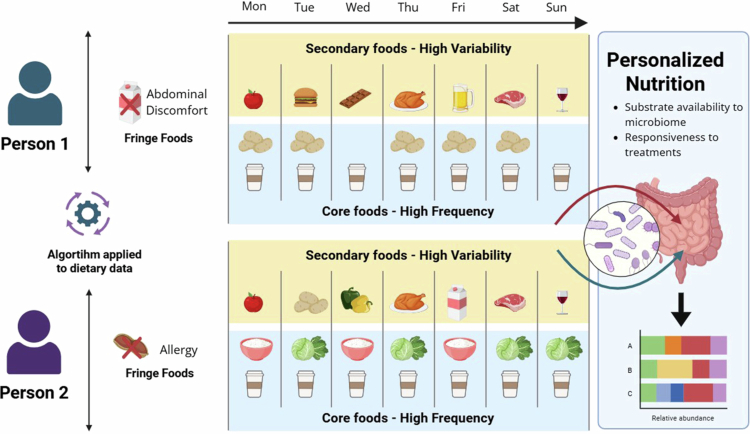
Food consumption at individual level can be divided into static dietary pattern corresponding to the “core foods”, while the dynamic dietary patterns corresponding to “secondary foods” are the more variable portion of food intake. These two groups can alter the substrate availability and responsiveness to treatments. In contrast, fringe foods are rarely consumed or deliberately avoided.

Recent reviews demonstrated that dietary patterns such as the Mediterranean diet, plant-based diets, ketogenic diets, and Western dietary patterns affect the gut microbiota composition.^14-16^ Therefore, these dietary patterns were not included in our original search strategy. Instead, our focus was on identifying studies that could inform the characterization of specific foods within static and dynamic dietary patterns. This perspective moves beyond generalized dietary patterns allowing for more granular dietary characterization when investigating microbiome-related outcomes or conducting dietary interventions. Figure 2 illustrates the difference between static and dynamic dietary patterns, and how core and secondary foods fit within these dietary patterns. Fringe foods can impact the gut microbiome and serve as triggers for GI symptoms, including abdominal discomfort, alterations in stool frequency, and acute changes in microbiome composition and diversity.

The following examples illustrate how static and dynamic dietary patterns can be integrated into various study designs to achieve specific research objectives.

1) **N-of-1 randomized crossover trial:** To characterize individual microbiome responses (multiple measures of stool samples) through repeated, systemic substitution of secondary foods while core foods remain stable. Randomization of the substitution sequence across repeated measures control temporal bias and microbiome variability within the individual.
2) **Cross-sectional study:** To examine how static and dynamic dietary patterns differ across individuals with microbiome-mediated diseases.
3) **Randomized crossover trial:** To test causal microbiome effects of different dynamic dietary patterns while maintaining individual static dietary pattern after a washout period.
4) **Randomized parallel trial:** To evaluate if changes in dynamic dietary pattern influence symptoms in patients with microbiome-mediated diseases.
5) **Prospective longitudinal cohort:** To estimate associations between consumption of the top 30 core foods and the frequency of gastrointestinal symptoms in patients with microbiome-mediated diseases.

**Figure 3.**
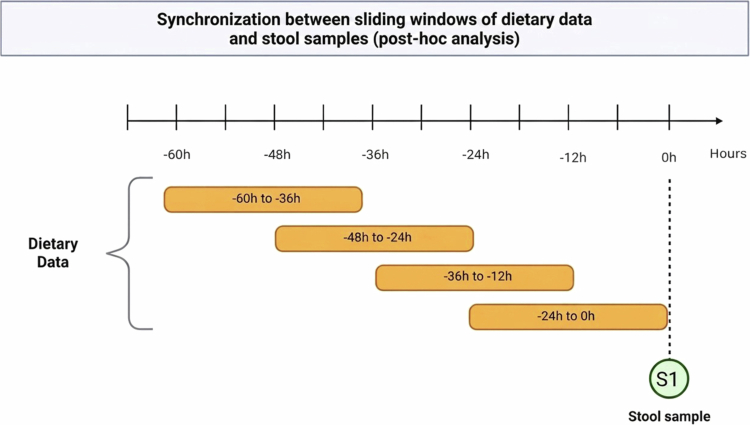
Hypothetical study design illustrating the sliding-windrow approach, used in post-hoc dietary analysis. The horizontal axis represents hours prior to each stool sample collection (t = 0), indicated by green circle, anchoring dietary windows an individual’s physiological event rather than to calendar days. Orange blocks represent overlapping 24-hour dietary assessment windows, each offset by 12 hours, constructed post-hoc to test which exposure period best predicts microbiome composition at the time of sampling.

Figure 3 depicts a longitudinal study design for a single individual, where each row represents a 24-hour retrospective sliding window of diet data for a single individual. This approach accounts for potential digestion lag times and gastrointestinal transit variability; a fixed-time window would not fully capture the different transit rates associated with the specific composition of the foods consumed. This design is applicable to randomized clinical trials, N-of-1 studies, and longitudinal cohort study. The proposed synchronization can reduce DNA sequencing costs while permitting trajectory analysis. However, participant burden and costs may be higher than in previous study designs that collect stool samples and dietary data only at baseline and endpoint.

## Discussion

This review introduces the concept of static and dynamic dietary patterns to improve the precision of diet-microbiome research. We summarize previous application of these concepts, identify key methodological gaps, and propose new strategies for applying static and dynamic dietary patterns at the individual level. Finally, we outline methodological considerations to help researchers more meaningfully evaluate how dietary exposures affect gut microbiome outcomes.

The concept of core foods was often used in population-based studies, with fewer studies addressing secondary foods. Most included studies involved large sample sizes and employed objectives and methods designed to capture a broad range of foods for analyzes of nutritional composition, chemical contaminants, or overall population-level consumption. Building on these population-based concepts, we extend Taylor et al. framework^31^ for dynamic food consumption by proposing food-level approaches to operationalize eating behavior at the individual level. This extension places greater emphasis on dietary assessment methods. Notably, only two out of 15 of the included studies used food frequency questionnaires alone to analyze the consumption.^43^^,^^48^ This approach can effectively characterize usual intake but is not the optimal approach to capture daily variation for gut microbiome research, especially during interventions. A key methodological limitation of long-term dietary assessment methods is their inability to provide sufficient granularity to identify core and secondary foods at the individual level. Strengths and limitations of dietary assessment tools should be evaluated in relation to specific research objectives and the hypothesized timing of gut microbiome responses.

Distinguishing between static and dynamic dietary patterns has significant implications for microbiome-targeted diet intervention strategies. Static patterns provide a foundational background that likely influences baseline microbial structure and metabolic activity. While static patterns (i.e. core foods) are likely to be deeply ingrained and difficult to modify, targeting them may result in more profound microbial impact. In contrast, dynamic dietary patterns may offer more feasible, but possibly more transient, targets for intervention. Regardless, this framework allows researchers to interpret short-term dietary shifts within the context of an individual’s underlying dietary background.

**Figure 4.**
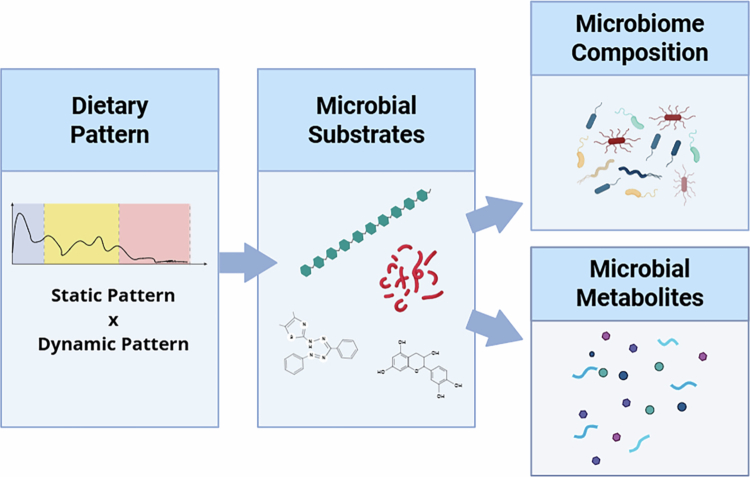
Conceptual framework linking dietary intake to gut microbiome composition and metabolic outputs. Static and dynamic dietary patterns determine the types of nutrients that escape host digestion and reach the colon. These substrates, as microbiota-accessible carbohydrates (e.g., dietary fibers and resistant starch), polyphenols, and unabsorbed proteins, serve as energy sources for the gut microbial community. The availability and accessibility of these substrates affect microbial composition and diversity, influencing taxonomic structure, microbial abundance, and community stability. Microbial metabolism subsequently generates functional outputs, including short-chain fatty acids (SCFAs), secondary bile acids, and other microbial metabolites that influence host physiology and intestinal barrier integrity.

Our proposed framework introduces principles for implementing personalized nutrition to improve health. Distinguishing static (consistently consumed) and dynamic (change over time) dietary patterns increase the accuracy of the dietary data in gut microbiome research by allowing the identification of short-term microbial responses to dietary changes. This structure also accommodates interindividual variability in diet–microbiome relationships, recognizing that dietary exposures may not cause uniform responses across individuals (Figure 4). We emphasize the importance of selecting validated dietary assessment instruments appropriate to the study objective to support data quality and reproducibility. The proposed framework provides a structured approach that can inform the development of practical research and clinical tools. An additional strength of the review includes the integration of evidence-based frameworks from nutrition and behavioral science to explicitly incorporate eating behavior into gut microbiome research, besides using the SANRA approach to guarantee the high quality of paper.^38^ This review also has limitations, such as the abstract screening was conducted by a single reviewer, which may introduce selection bias. However, studies selected for full-text review were evaluated by all authors. The proposed framework has not yet been empirically validated at the individual level.

Conceptualizing diet as static and dynamic components provides a useful framework to operationalize these definitions and investigate the interplay of usual diet with intervention on the gut microbiome. The proposed considerations outline potential directions for research design and methodological decision-making when assessing diet–microbiome relationships. Future studies can develop and validate operational criteria (e.g., consumption frequency, time windows, intake thresholds, food grouping) with criteria tailored to the research question and target health outcome. Continued methodological development and empirical validation will be needed to refine these approaches and evaluate their use for different study designs and patient populations.

## Supplementary Material

Supplementary Material Static Dynamic Supplementary Material.docx

## Data Availability

No data were generated nor analyzed for the manuscript.

## Generative artificial intelligence (AI) use statement

After completing a full manuscript draft, the author(s) used ChatGPT 5.2 for language refinement only. After using this tool, the authors reviewed and edited the content and are taking full responsibility for the content of the publication.
